# Semantic meaning enhances feature-binding but not quantity or precision of locations in visual working memory

**DOI:** 10.3758/s13421-024-01611-x

**Published:** 2024-07-30

**Authors:** Tomer Sahar, Nurit Gronau, Tal Makovski

**Affiliations:** 1 Department of Psychology and Education, The Open University of Israel, Ra’anana, Israel; 2 School of Psychological Sciences & The Institute of Information Processing and Decision Making, University of Haifa, Haifa, Israel

**Keywords:** Real-world items, Meaningless items, Estimation task, Spatial location

## Abstract

Recent studies showed that real-world items are better remembered in visual working memory (VWM) than visually similar stimuli that are stripped of their semantic meaning. However, the exact nature of this advantage remains unclear. We used meaningful and meaningless stimuli in a location-reproduction VWM task. Employing a mixture-modeling analysis, we examined whether semantic meaning enables more item locations to be remembered, whether it improves the precision of the locations stored in memory, or whether it improves binding between the specific items and their locations. Participants were presented with streams of four (Experiments 1 & 2) or six (Experiment 3) real-world items, or their scrambled, meaningless counterparts. Each item was presented at a unique location, and the task was to reproduce one item’s location. Overall, location memory was consistently better for real-world items compared with their scrambled counterparts. Furthermore, the results revealed that participants were less likely to make swap errors for the meaningful items, but there was no effect of conceptual meaning on the guess rate or the precision of the report. In line with previous findings, these results indicate that conceptual meaning enhances VWM for arbitrary stimulus properties such as item location, and this improvement is primarily due to a more efficient identity-location binding rather than an increase in the quantity or quality (precision) of the locations held in memory.

## Introduction

Studies of visual memory have extensively investigated both the breadth and capacity limits of visual memory. Classical as well as more recent long-term memory (LTM) studies found that observers can remember a vast number of items and their features or states, to the extent that, effectively, no upper bound of capacity was established (e.g., Brady et al., 2008, 2011; Konkle et al., 2010a, b; Shepard, 1967; Standing, 1973). Those studies used meaningful items such as real-world objects and scenes as memoranda, lending support for the important role of semantic meaning in visual LTM (e.g., Brady et al., 2019; Konkle et al., 2010a, b; Kouststaal et al., 2003; Kramer et al., 2023; Reder et al., 2013; Shoval et al., 2023a). Indeed, when real-world objects were stripped of their semantic meaning through scrambling and distortion, visual memory capacity was shown to be dramatically reduced (Shoval et al., 2023b).

In an attempt to avoid LTM influences, however, classical visual working memory (VWM) studies have typically employed “low-level” stimuli such as colors, line orientations, and meaningless matrix patterns (e.g., Luck & Vogel, 1997; Phillips, 1974). In contrast to LTM findings, these studies have traditionally demonstrated a highly limited capacity system in VWM (e.g., Cowan, 2010; Luck & Vogel, 2013; Oberauer et al., 2016; Zhang & Luck, 2008, 2009). However, recent research has indicated that VWM performance is actually enhanced when the memoranda carry semantic meaning (e.g., Asp et al., 2021; Brady & Störmer, 2022; Brady et al., 2016; Chung et al., 2023a; Conci et al., 2021, 2023; Sahar et al., 2020; Shoval & Makovski, 2022).

For example, Sahar et al. (2020) compared VWM and the metamemory of meaningful and meaningless stimuli. The meaningful stimuli comprised images of real-world objects (Brady et al., 2008), while the meaningless stimuli were the same images subjected to a simple image manipulation, preserving most visual properties while reducing their meaning (see Makovski, 2018). The results revealed that participants better remembered semantically meaningful items than meaningless items with higher confidence. The disadvantage of meaningless items was observed in following studies regardless of whether the items were “lightly” (e.g., Makovski, 2018) or “heavily” (Stojanoski & Cusack, 2014, Fig. 1b) distorted (Brady & Störmer, 2022; Shoval & Makovski, 2022). Another study (Brady & Störmer, 2022) showed that meaningful items were better remembered in both serial and simultaneous presentation than blobs of colors and distorted items (see also Chung et al., 2023a). Supported by past findings of a larger CDA (contralateral delayed activity) component for real-world items than color patches (Brady et al., 2016), the authors stipulated that memory capacity is boosted for meaningful items since they receive in-depth encoding and stronger active maintenance in VWM (see Asp et al., 2021; also, Brady et al., 2019, in LTM). While a follow-up study (Thibeault et al., 2023) was not able to pinpoint the precise locus of improvement (i.e., encoding, maintenance, or retrieval), it demonstrated an improvement in capacity estimates (Cowan’s *K*) of meaningful stimuli compared with meaningless items. The authors suggested it is the conceptual meaning rather than the visual complexity of the items that drives a VWM advantage for real-world object identities.

**Fig. 1:**
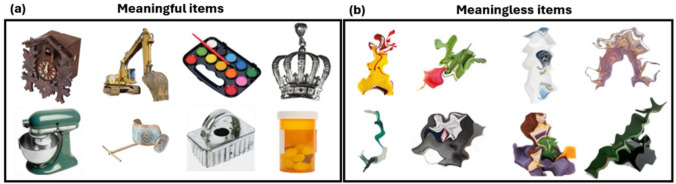
Examples of stimuli from all experiments. **a** Examples of intact, meaningful items. **b** Examples of distorted, meaningless items. (Color figure online)

The *semantic meaning* of an (visual) item can be defined from various perspectives. Here, we consider the semantics of an object as typically derived from its basic-level category—namely, the level at which the item can be labeled. It is important to note that a label or a name encapsulates a set of critical features defining the item (Rosch, 1975; see Gasparri & Marconi, 2021), yet the manner in which these features contribute to visual memory remains largely unclear. That is, how idiosyncratic nonvisual information (i.e., conceptual meaning) contributes to the enhancement of visual information in memory is quite remarkable and not yet fully explained.

There are several, not mutually exclusive explanations for the advantage of semantic meaning in VWM. In some studies, semantic meaning has been described as a “hook” or a “scaffold” for visual detail (e.g., Chung et al., 2023a; Konkle et al., 2010a, b). More specifically, it was suggested that real-world objects are stored in memory at both feature and object-based levels, with the latter linked to conceptual knowledge. This hierarchical organization provides a valuable structure (or “scaffold”) for low-level visual features and a higher-dimensional representation, thereby enhancing VWM (e.g., Chung et al., 2023a; Wyble et al., 2016). From a neurophysiological perspective, meaningful items may activate brain regions that support the formation of more vivid visual memories compared with meaningless stimuli (e.g., Asp et al., 2021; Brady & Alvarez, 2015; Shoham et al., 2022). Relatedly, some argued that prior knowledge about an object enables the “chunking” of information, thereby reducing capacity limitations and enabling observers to maintain more features and information (e.g., Brady et al., 2009; Conci et al., 2021).

Previous existing knowledge is also tied to stimulus familiarity, with familiar items benefiting from better consolidation of items into VWM (Blalock, 2015; Shoval & Makovski, 2021; Xie & Zhang, 2017, 2018). In alignment with this perspective, other views have proposed that familiarity contributes to visual memory performance by freeing attentional resources to encode more visual information and facilitate feature-to-feature binding (Popov & Reder, 2020; Popov et al., 2022; Reder et al., 2013). Another way in which meaning might play a role in VWM is through the engagement of verbal LTM or labeling. When participants are given the option to use a term that describes the visual stimulus (i.e., a label), VWM performance improves both in terms of quantity and quality compared with a condition involving meaningless articulatory suppression (Souza & Skóra, 2017). Labeling has been shown to boost VWM performance by allowing to maintain and protect a detailed representation in VWM (Overkott & Souza, 2022, 2023). However, conceptual meaning does not *necessitate* explicit verbal encoding. It was suggested that meaningful visual items might activate some form of conceptual, and perhaps prototypical, knowledge irrespective of verbal encoding (Bae et al., 2015; Markov & Utochkin, 2022; Sahar et al., 2020; Souza et al., 2021). Given the intimate connection between perceptual and conceptual information in everyday objects, it is often highly challenging to separate the two and isolate “perceptual” memory per se (see Konkle et al., 2010a, b, in LTM). Notably, the type of visual information being tested might affect one’s conclusions. For instance, A feature might be easier to encode and consolidate into VWM if it aligns with preexisting knowledge (Bae et al., 2015; Olsson & Poom, 2005), or if it is diagnostic of the object’s nature/identity (e.g., distinguishing between a green and yellow banana, or recognizing the colors of a familiar flag; e.g., Conci et al., 2021). In the present study, we therefore asked whether conceptual knowledge could affect memory for visual details that are not associated with an item’s core essence. Specifically, we investigated whether meaning could enhance VWM of an *arbitrary* object feature, such as its spatial location.

If meaning does indeed enhance location memory performance, we aimed to observe whether this enhancement operates in terms of memory resolution or other factors. The results of Chung et al. (2023a), demonstrating that memory for an arbitrary color is enhanced for meaningful items, already suggest that the influences of meaning might not be restricted to the item’s identity. Yet it is not clear whether this result can be generalized to other dimensions that are not directly related to the item’s intrinsic features. Critically, in the context of the current study, location serves as an arbitrary feature that is independent of the item’s identity.

**Fig. 2:**
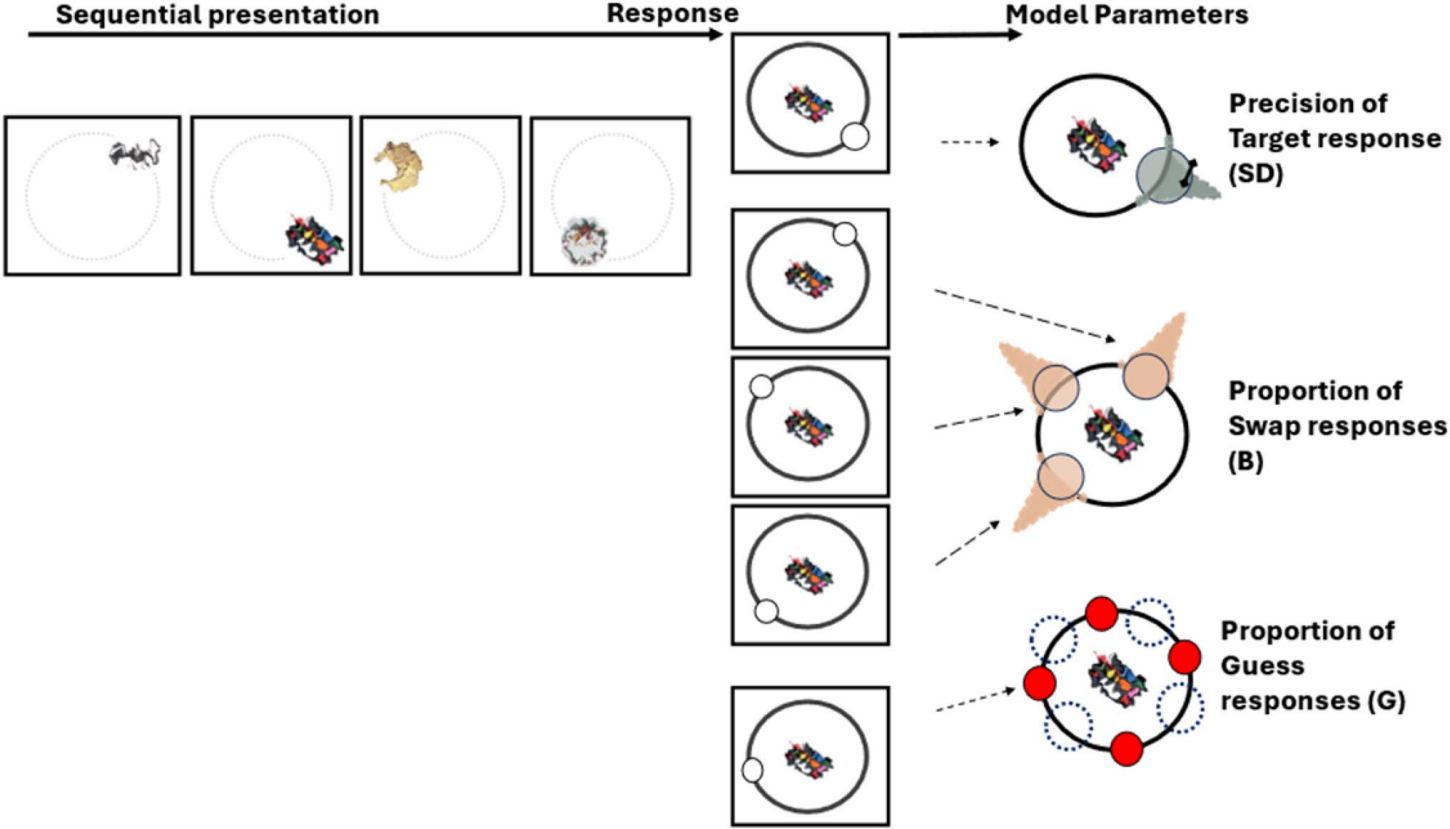
Schematic illustration of a trial containing meaningless items and the mixture-modeling approach with the swap model (Bays et al., 2009). (Left) **Presentation sequence:** Each trial consists of four items presented sequentially, each presented in a distinct location around the perimeter of a circle (see details below and Fig. 3). (Middle) **Response:** In the response phase, participants are asked to reproduce the target location (i.e., the location of the item appearing inside the circle) by continuously adjusting a probe (small white circle) to the target location on the perimeter of the outer circle. (Right) **Correspondence of responses to the model parameters:** Responses in the near vicinity of the correct target location are classified as *target* responses (marked in gray); the *precision* of response (SD) is estimated from the spread of responses. Responses at the location of nontarget items (marked in orange) are classified as *swap* responses. Unrelated responses (marked in red) are classified as guess responses. (Color figure online)

Furthermore, testing spatial location memory is essential because many researchers have argued that location constitutes a unique feature, playing a key role in attention, perception, and VWM (e.g., Golomb et al., 2014; Lamy & Tsal, 2001; Makovski 2016; Pertzov & Husain, 2014; Treisman & Gelade, 1980), specifically in the binding of different features together (Oberauer & Lin, 2017; Schneegans & Bays, 2017; but see Li et al., 2022). The goal of the study was thus to test the effects of meaning on spatial memory, by comparing performance for meaningful (i.e., intact, real-world) with meaningless (i.e., scrambled) objects (see Fig. 1). Furthermore, using a mixture-modeling approach (Bays et al., 2009), we were able to test which parameters were specifically affected by the item’s meaning (for a similar approach, see Markov & Utochkin, 2022; Popov et al., 2022). There are several possible outcomes to the mixture-modeling approach (see graphical illustration in Fig. 2, right): (1) An effect on the SD parameter (i.e., smaller SDs for meaningful items) would suggest better location memory precision. That is, meaning creates a more precise location representation. (2) An effect on the guess rate would suggest that meaning boosts the number of locations stored in VWM. (3) Fewer swap errors for meaningful items would suggest a stronger linkage between arbitrary spatial location and identity. Note that the combination of guess-rate and the swap-rate indexes the number of correct object locations stored in memory: If meaning boosts the number of locations stored in VWM, then we should expect a higher rate of correct target responses given the item’s identity (i.e., fewer errors due to guesses and swaps) for the meaningful stimuli.

It is also noteworthy that any combination of these outcomes is possible and that these possibilities do not necessarily refute the suggested theories and accounts. This is because the theories can be modified to accommodate any of the (parameters) outcomes. For example, if all items have an accessible memory signal, but it is weakly bound to their specific locations, “increased capacity” could be expressed as fewer swap errors. The current study, therefore, was not designed to pit the different theoretical views against each other, but rather to ask *which* aspects of memory performance (i.e., parameters in the model) would be mostly affected by stimulus meaning.

To preview our results, Experiment 1 found that meaning had a large effect on VWM performance that translated to an effect on *swap* errors, while there was no effect on the guess rate or the precision rate (SD). Experiment 2 replicated Experiment 1’s results, even though it included masks that impaired overall performance, negating the possibility that the lack of an effect on precision might have resulted from a ceiling effect. Experiment 3 further tested VWM for a larger memory set size, and yet the main results remained the same: Meaning reduced swap errors but did not affect the guess rate or the precision factor. Since all experiments share the same general method, we report them all collectively.

## Method

The stimuli, tasks, data, and analyses are available at the Open Science Framework (https://osf.io/za3bm).

### Sample size

We adopted an open-ended Bayesian design (Schönbrodt & Wagenmakers, 2018). We set the minimal sample size at 20 participants. This sample size allows us to detect a large effect (Cohen’s *d* = .7, *p* = .05) with 85% statistical power. Due to the exploratory nature of Experiment 1, we allowed a larger sample size to make sure we could detect an effect (if any) on each parameter and adopted a stricter criterion to stop collecting data (i.e., BF > 100 for H_0_ or H_1_). In Experiments 2 and 3, given the observed effects of Experiment 1, we adopted a looser criterion for stopping the collection of data—when a two-sided Bayesian paired-samples *t* test for the average absolute error (i.e., meaningful vs. meaningless) showed a BF above 3 (for either H_0_ or, H_1_). The mixture modeling was performed only after the data collection was completed. In the results section BF_10_ denotes the Bayes factor in favor of a difference (i.e., an effect present) while BF_01_ denotes the Bayes factor in favor of no difference (i.e., the absence of an effect). The two measures are reciprocal and easily interchangeable: ${BF}_{10}= \frac{1}{{BF}_{01}}$

### Participants

Participants in all experiments were the Open University of Israel undergraduate students who took part in the experiments for course credit (age range: 18–40 years). All signed an informed consent, and had normal or corrected vision, normal color vision, and lacked any neurological or attention deficits. The study was approved by the ethics committee of the Education and Psychology department at the Open University. Experiment 1 tested 35 participants (18 females, 10 males, seven other/not responded, mean age = 29.4 years), Experiment 2 tested 24 participants (22 females, two males, mean age = 26 years), and Experiment 3 tested 25 participants (12 females, 13 males, mean age = 27.3 years).

### Materials

The task was implemented with PsychoPy (Version 2020.1.3; Peirce et al., 2019), running on a Standard PC with a 23.5-in. LCD Eizo Foris monitor (1,920 × 1,080, 120-Hz refresh rate). Analyses were performed using JASP (Version 0.15, JASP team, 2021) with the standard prior (Caucy~.707). Mixture-modeling analyses and model comparisons were performed using the MemToolbox package (Suchow et al., 2013). All stimuli appeared against a white background (RGB = [0, 0, 0]). All experiments included a starting fixation display (a black cross, 50 pix). Stimuli appeared on an invisible circle with a radius of 380 pix (11.4°, except in Experiment 3 in which it was visible and appeared in black (RGB = [255, 255, 255]). Each image size was 160 × 160 pix (4.8° × 4.8°), except for Experiment 3, in which the image size was 100 × 100 pix (3° × 3°). The image set (for each participant) included 600 randomly sampled intact images of everyday objects from a previously published set (which contains 1,600 images: Brady et al., 2008). The distorted images were a subset of 600 scrambled versions of intact images, also sampled from the larger pool (of 1,600 scrambled images). Thus, images in the two conditions were roughly matched across participants. Scrambling was performed by diffeomorphic transformations (Stojanoski & Cusack, 2014) that distorted the images’ meaning while keeping most of the visual statistics intact (Brady & Störmer, 2022; Shoval et al. 2023b; Shoval & Makovski, 2022). An image of a multicolored circle was presented before the first and after the last items so each trial sequence started and ended with the colored-squares image. The mask image was a circle filled with multicolored squares, of the same dimensions as the images. The locations of the memory items were achieved by presenting the stimuli in a radial orientation ranging from 1° to 360° in steps of 1°. Each image (160 × 160 pix) appeared in a random, unique position with the restriction of a minimum distance of 30° between the images. Participants responded to the memory-location task by using the mouse to adjust a response probe to the target’s location. The response probe was a white circle (radius Experiments 1 and 2: 80 pix, 2.4°; Experiment 3: 50 pix, 1.5°) that appeared on a visible black circle (RGB = [255, 255, 255]), of the same dimensions as the objects presented during encoding. After the response, participants received written feedback (“your error is X degrees”; RGB = [0,0,0], Helvetica, 30 pix) presented inside the response circle, 80 pix below the probe. In addition, the correct target location appeared on the circle with a red “X,” and the participant’s response appeared simultaneously with a blue “O” (40 pix).

### Procedure

#### Experiment 1

**Fig. 3:**
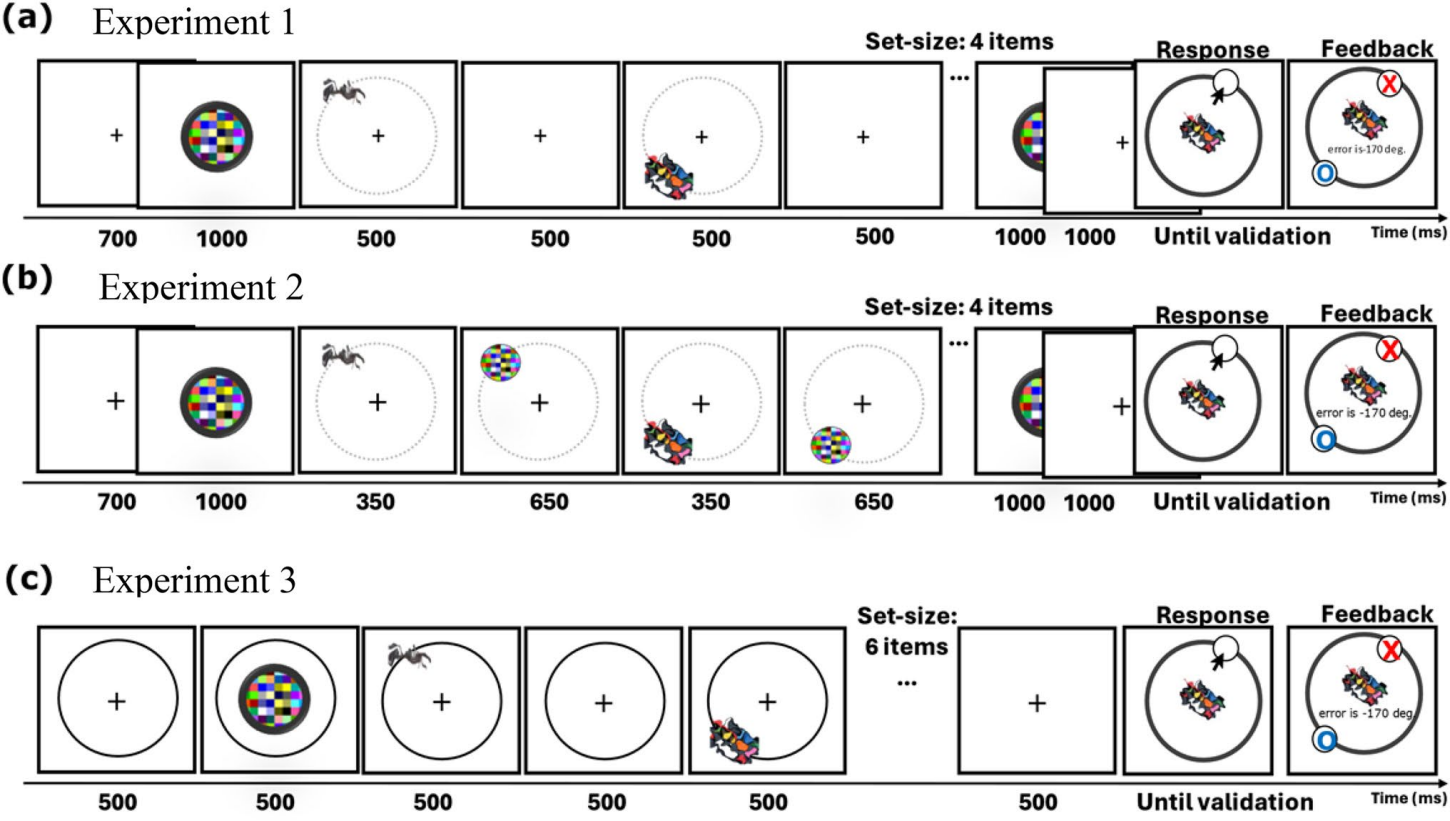
Schematic illustration of the trial’s sequence in Experiment 1 (**a**), 2 (**b**), and 3 (**c**). *Note.* Items and displays are not scaled to their real size; Experiments 3 used smaller images. (Color figure online)

The participants’ task was to remember the location of four images so they could adjust the probe to match the target’s location. Four unique images, either intact (meaningful) or distorted (meaningless), were randomly sampled, and each image was used only once throughout the experiment. Each trial began with a 700-ms fixation display—a black plus sign (50 pix) that remained visible throughout the trial. The first colored circle appeared at the center of the screen for 1,000 ms. Then, each of the four images appeared in their location, in isolation, for 500 ms, followed by a 500-ms blank screen. After the presentation of all four images, the colored-circle image appeared again at the center of the screen for 1,000 ms. Then, after another blank screen of 1,000 ms, the target image appeared at the center of a black circle (drawn in the same radius as the presentation sequence). The target was one of the four images from the trial’s sequence and its serial position was evenly and randomly assigned across trials. Once the participant clicked with the mouse on the circle, the response probe, a white circle, appeared at the clicked location on the circle’s perimeter. The participant adjusted the probe (using the mouse) and validated her response by pressing the spacebar key on the keyboard. Notably, there was no time limit and participants could not validate their response without moving the mouse first. Afterward, the feedback display appeared for 1,000 ms (Fig. 3a). Importantly, 96 trials of meaningful items were randomly intermixed with 96 trials of meaningless items (with each trial consisting of same-type objects only). For practice, participants performed a few test trials to ensure they understood the task. These trials were not recorded, and their images were not included in the experimental trials.

#### Experiment 2

The design, procedure, and stimuli were the same as in Experiment 1, except for the following changes (see Fig. 3b). Each item appeared for 350 ms, followed by a mask image of the same object size that appeared immediately after each of the objects in their location, for 650 ms. This was done in order to replicate Experiment 1’s results under more demanding encoding conditions.

#### Experiment 3

To rule out the possibility that the lack of an SD (precision) effect was due to ceiling performance, as well as to directly test whether semantic meaning allows more locations to be stored in VWM for meaningful items, the memory set size was enlarged to six items. Increasing the number of potential target locations necessitated the use of smaller items and a larger circle, thus stimuli size was reduced to 100 × 100 pix. Note that this adjustment also creates more space for an SD effect. The black circle, serving as a landmark, remained visible throughout the trial while the items appeared on its perimeter. Finally, to shorten the experiment, the initial fixation display and the final fixation display were presented for 500 ms only. The colored circle at the end of the sequence was not presented (see Fig. 3c). There was a total of 180 trials, 90 in each condition (i.e., meaningful/meaningless). In all other respects, the experiment was identical to Experiment 1.

### Mixture-model analysis

On each trial, we calculated the error magnitude, that is, the difference between the adjusted probe location and the target location. The error magnitude ranged from ±180°. Modeling was performed with MemToolbox (Suchow et al., 2013) and we used the swap model (Bays et al., 2009). This model received the lowest AIC and BIC scores (corrected Akaike information criteria and Bayesian information criteria, respectively) against the standard model (Zhang & Luck, 2008), and the variable precision model (Van den Berg et al., 2012). The swap model consists of three parameters: (1) Standard deviation (*SD*): The standard deviation of target responses, reflecting the precision of the target’s report in degrees. (2) Proportion of swap responses (*B*): The proportion of trials where participants mistakenly reported the location of one of the distractor items instead of the target item, (3) Proportion of guess responses (*G*): proportion of trials with a large magnitude error originating from a uniform distribution, and unrelated to any of the distractors’ location. For each participant, each condition (i.e., meaningful, meaningless) was modeled separately. Each of the three parameters was aggregated across participants to create an overall model for each condition.^1^ In general, the model computes the *SD* based on errors around 0 (target). This estimate of *SD* is then applied around the center of each distractor. This holds that participants had the same “target” precision but only misreported the location of the item. Because the items were widely separated, low-precision reports (within the range of the estimated *SD*) would fall within the appropriate item. Unrelated location reports would fall between items, and most likely would be classified as “guesses.”

## Results

**Fig. 4:**
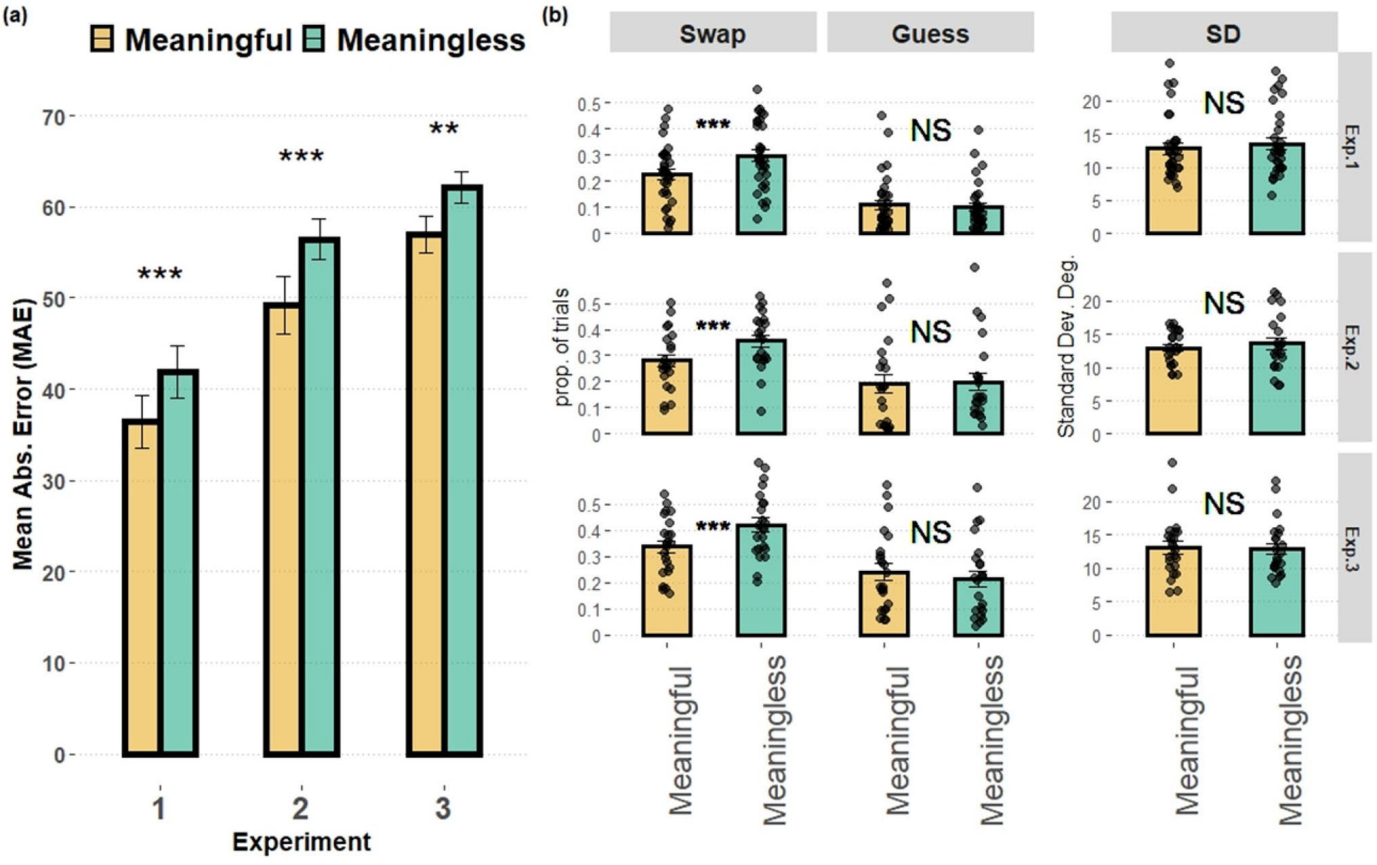
Results in the three experiments. **a** Overall spatial memory performance: The mean absolute error (MAE) as a function of stimulus condition and experiment. **b** Model parameters: In rows, the results for each experiment. In columns, parameter values: proportion of swap rate, proportion of guess rate, and the standard deviation in degrees (*SD*) as a function of stimulus condition. Black circles represent individual data. Error bars denote 1 *SEM*. **p < .01. ***p < .001. NS = *p* > .05

Results from all experiments are detailed in Fig. 4. To quickly overview, as can be seen in Fig. 4a, the model-free mean absolute error (MAE) shows an effect of meaning across all experiments: a smaller overall magnitude of errors for meaningful than for meaningless items. As can be seen in Fig. 4b, depicting the mixture-model parameters, only the swap parameter was affected by meaning, as trials containing meaningful items had fewer swap errors.

### Experiment 1

Three participants were excluded from the analysis (two for large MAE > 80 °, and one participant’s data did not converge). The removal of these data did not affect any of the conclusions.

First, to ensure that meaning influenced memory performance, we compared the MAE magnitude between meaningful-items and meaningless-items trials. A two-sided paired-samples *t* test revealed a significant difference between the two conditions, *t*(31) = 4.18, *p* < .001, *d* = .73, and a substantial Bayes factor of BF_10_ = 125 was in accord with the stopping rule. Next, we compared each of the three model’s parameters, between meaningful and meaningless trials. Only the swap error parameter (*B*) was significantly different between the conditions, *t*(31) = 4.4, *p* <.001, *d* = .79, BF_10_ = 274. We did not observe a significant difference in the SD parameter, *t*(31) = 1.1, *p* = .242, *d* = .21, BF_01_ = 2.77, or in the guess rate parameter, *t*(31) = 0.5, *p* = .613, *d* = .09, BF_01_ = 4.69.

### Experiment 2

Two participants were excluded from the analysis (MAE > 80 ) and the removal of these data did not affect any of the conclusions.

Overall performance was indeed worse in Experiment 2 than in Experiment 1 as seen in the MAE, independent samples, *M* = 52° vs. 39°, *t*(52) = 3.43, *p* = .001, *d* = .95. Importantly, once again, there was an effect of meaning on the MAE, *t*(21) = 3.75, *p* = .001, *d* = .8, and the Bayes factor of BF_10_ = 31 was in accord with the stopping rule. Comparing the model parameters, we observed the same results as in Experiment 1. The swap error parameter (*B*) was significantly different between the conditions, *t*(21) = 3.76, *p* = .001, *d* = .8, BF_10_ = 31. We did not observe a statistically significant difference in the SD parameter, *t*(21) = .7, *p* = .468, *d* = .15, BF_01_ = 3.5, nor in the guess rate parameter, *t*(21) = 0.4, *p* = .639, *d* = .1, BF_01_ = 4.04.

### Experiment 3

Two participants were excluded from the analysis (one participant’s data did not converge and one participant’s *SD* estimate was inflated, *SD* > 89°). The removal of these data did not affect any of the conclusions.

Increasing memory set size had a large effect on the overall memory performance compared with Experiment 1, as evident in the MAE, nonparametric independent samples Welch, *M* = 59°vs. 39°, *t*(48.6) = 6.2, *p* < .001, *d* = 1.6. Nevertheless, we observed the same pattern of results as in our previous experiments. The overall effect of meaning was significant, as evident in the MAE magnitude, *t*(22) = 3.09, *p* = .005, *d* = .64, and the Bayes factor of BF_10_ = 8.3 was in accord with the stoppage rule. Still, once again, the same pattern emerged: a significant difference for the swap error parameter, *t*(22) =3.14, *p* = .005, *d* = .655, BF_10_ = 9.2, but not for the SD parameter, *t*(22) = .18, *p* = .852,* d* = .03, BF_01_ = 4.4, nor the guess rate, *t*(22) = .79, *p* = .435, *d* = .16, BF_01_ = 3.4.

### Location memory precision

Given the idea that spatial location has a special status, in this analysis, we focused on the potential difference (or lack of) between experiments in the precision of location memory. An across-experiment analysis with the SD parameter as the dependent variable (repeated-measures ANOVA: meaningful/meaningless as a within-subject factor and experiment as a between-subjects factor) did not show statistically significant differences in the precision of spatial location by the item meaning, *F*(1, 74) = .01, *p* = .917, nor by its interaction with the experiment, *F*(2, 74) = .75, *p* = .473. Bayesian RMANOVA, with the same factors, corroborated that the precision parameter was unaffected by stimulus meaning (BF_01_ = 8.4), and did not interact with experimental conditions (BF_01_ = 8.9), noting an overall high degree of precision (*M* = 13.7°). Thus, semantic meaning does not affect precision, which seems high to begin with.

## General discussion

The present study examined how items’ conceptual meaning affects VWM performance.

While traditional VWM research used simple stimuli to reduce LTM involvement, we manipulated the conceptual meaning of visually complex items, to directly examine LTM involvement in VWM. Specifically, we asked which aspect of memory is improved by conceptual meaning and whether meaning allows observers to better retain an arbitrary visual property of the stimuli, such as its spatial location. In three experiments, we compared two types of stimuli: Meaningful real-world items and scrambled, meaningless items. The scrambling procedure preserved most of the visual statistics of the stimuli while stripping them of their semantic meaning (Brady & Störmer, 2022; Chung et al., 2023a; Sahar et al., 2020; Shoval & Makovski, 2022; Shoval et al., 2023a, b; Starr et al., 2020); thus, the two stimulus sets mainly differed in their conceptual distinctiveness (but see reservation below). Consequently, when observers were exposed to the scrambled, meaningless items, they could not readily access the items’ conceptual information, instead *primarily* relying on their visual features.

Experiment 1 revealed a large overall benefit in the location memory of meaningful items.

When the memory performance was decomposed into parameters using a mixture-modeling analysis (Bays et al., 2009), we observed that the benefit was driven only by fewer swap errors, not by better precision or by a smaller guess rate. That is, the advantage of meaningful items resulted from the fact that observers were more frequently correct about the item’s location *given* its known identity (i.e., the memory probe). The results also revealed high precision in reporting the target’s location along with a low guess rate that might have concealed an effect in these parameters. Therefore, in Experiment 2, we shortened the encoding duration and masked each item. Despite an increase in the overall magnitude of errors, we observed again a large location memory benefit for meaningful items and the same pattern of results when decomposing the findings to the model parameters: Fewer swap errors for meaningful stimuli, along with no difference for both the SD and the guess-rate parameters. Experiment 3 used a larger set size—six items instead of four items. Once again, although this time smaller in magnitude, we observed an advantage for meaningful items. Consistently, as in the previous experiments, fewer swap errors were found for meaningful stimuli, and no effect was observed for the SD and the guess-rate parameters. Collectively, we showed that the semantic meaning benefit was driven by fewer swap errors for meaningful stimuli rather than by improved precision or fewer location guesses.

Three main conclusions can be drawn from this study. First, overall memory performance, as measured by the model-free mean absolute error magnitude (MAE), clearly showed that meaning improved memory performance, specifically for spatial memory of an arbitrary location. This result extends past findings demonstrating a role for conceptual meaning in VWM (Asp et al., 2021; Conci et al., 2021; Overkott & Souza, 2023; Sahar et al., 2020; Shoval & Makovski, 2022; Starr et al., 2020; Brady & Störmer, 2022). Furthermore, our findings demonstrate that memory not only enhances stimulus aspects related to core identity, but also to an arbitrary feature, such as location or color (Chung et al., 2023a). The findings that semantic meaning dramatically improves VWM performance may challenge the external validity of numerous findings from previous studies using “simple” (meaningless) stimuli.

Second, a deeper examination of performance reveals that the main factor affected by semantic meaning is feature binding, as participants made fewer location-identity (or location-shape) swap errors with meaningful than meaningless items. This finding suggests that the enhancement of retention capacity by semantic meaning might specifically relate to stimulus feature bindings, as suggested by Oberauer (2019). Our results are analogous to the findings of Markov and Utochkin (2022), who used a similar location reproduction task and analysis. In contrast to the current study, these authors used only real-world items (i.e., meaningful stimuli), for which they varied the conceptual distinctiveness in the memory array. That is, they contrasted same-category item trials (e.g., all apples, characterized by a low distinctiveness) with different-category item trials (e.g., an apple, a mug, and a rabbit, characterized by a high distinctiveness).

Most relevant to the current results, Markov and Utochkin (2022) consistently found that categorical similarity did *not* affect correct recognition rates of the item’s identity, and did not affect the overall precision or guess rate for spatial locations. Similarly to the present findings, more swap errors were observed under the low-distinctiveness (i.e., high similarity) than the high-distinctiveness (low similarity) condition. That is, observers frequently swapped the location of items within the same category, suggesting that conceptual similarity creates interference between items. Notably, however, items from the same conceptual category also shared a high degree of visual similarity, whereas, in the present study, the meaningless items varied in their perceptual features (albeit the scrambling procedure may have somewhat reduced their visual distinctiveness, relative to the meaningful objects). Nevertheless, despite these differences, the conclusions of both studies converge on each other: Observers tend to rely on conceptual meaning in reducing item-to-item interference when maintaining and retrieving stimuli’s locations in VWM. Note that similar results were obtained when examining the effects of conceptual meaning on memory for an arbitrary color—that is, an enhanced item-color binding for the meaningful objects (Chung et al., 2023a, b).

Lastly, the semantic meaning of the items did not affect the degree of location memory precision, which remained relatively high across all three experiments. Though speculative, this high precision might reflect the special status of spatial location. A similar high precision was observed by Markov and Utochkin (2022), using a within- versus between-category manipulation. Furthermore, recent findings demonstrated that the recall of stimulus location (on a continuous report scale) yielded the highest precision and accuracy rates compared with orientation, color, and shape (Tomić & Bays, 2022). Thus, the inherently high accuracy and precision of spatial locations in this, as well as other studies, might have obscured a potential effect with the precision parameter. Alternatively, the absence of a semantic effect on spatial location memory may suggest that spatial location per se (“where”) is processed independently of semantic meaning (“what”), and therefore, the latter exerts little direct impact on it. However, such a segregated view may oversimplify matters, as findings have shown that semantics and real-world items improved the accuracy in spatial working memory change-detection tasks (Hu & Jacobs, 2021).

Despite clear behavioral evidence that conceptual meaning improved item–feature binding, the current study cannot provide a definitive underlying mechanism for this pattern of results. However, at least two explanatory models have nevertheless been suggested for the effects of conceptual meaning on memory. First, Popov and Reder (2020) provided a computational model that showed that contextual memory effects in word lists (e.g., recency, primacy) can be explained by the frequency (i.e., familiarity) of the stimuli. Given a limited pool of resources, the authors suggested that high-frequency words were better remembered due to their consumption of less attentional resources for encoding than low-frequency words. They concluded that the formation of a memory representation requires the use of resources proportionally to the item’s *memory strength* (i.e., reflecting as a robust and accessible representation). In the present study, one might consider meaningful items to be more “strongly” encoded due to their inherent familiarity, relative to the novel, scrambled items. Put differently, meaningful items have a stronger memory signal since they are heavily associated with prior knowledge. Consequently, they require less resources for their encoding, sparing more resources for the encoding of additional information, such as their location (see also Bellana et al., 2021; Reder et al., 2013). This idea is closely related to the notion that activating an LTM representation in VWM consists of prototypical information (like a common label, shape, and surface features) that strongly defines the object. Such information can increase the dimensionality of the representation while reducing the information entropy (i.e., the cost of processing every bit of visual information). Interestingly, consistent with the findings of our study, Popov et al. (2022) showed that word familiarity (operationalized by high vs. low frequency) affected the probability of location swap errors, but not the precision memory of the target location. Again, the fact that feature-binding processes are long known to require attentional capacity (e.g., Treisman & Gelade, 1980) is highly consistent with the resource-based account (Popov & Reder, 2020; Popov et al., 2022).

A different model, emerging from a signal detection perspective (target confusability competition [TCC]; Schurgin et al., 2020; see also Brady et al., 2024; Oberauer, 2023), suggests that swap errors occur due to a lower signal-to-noise ratio (i.e., lower *d′*). That is, visual similarity between items creates “confusability” between the target item and the foils. This is consistent with the current result as one may suggest that meaningful items have a stronger memory signal, on the account that they are more visually and a conceptually distinct. Although the scrambling manipulation maintained most low-level features and image statistics, the fact that local as well as global contours were largely blurred turned the meaningless items into somewhat less visually distinct from each other. Whether on a visual or a conceptual level, both types of distinctiveness (i.e., degree of similarity) can contribute to such pattern of errors (but see Tomić & Bays, 2022). Further research is needed to examine, for example, if similar (conceptually) meaningful items that strongly differ in visual properties indeed support a stronger memory signal.

To conclude, our findings show that spatial location memory, as an arbitrary visual property, benefits from the item’s conceptual meaning. This finding corroborates previous research, demonstrating how LTM involvement (long-term conceptual knowledge) enhances VWM performance (Asp et al., 2021; Chung et al., 2023a; Conci et al., 2021; Oberauer et al., 2017; Overkott & Souza, 2023; Sahar et al., 2020; Shoval & Makovski, 2022; Brady & Störmer, 2022). Critically, we show that meaning improves VWM for an item’s location by specifically enhancing the binding between objects and their features similar to previous findings (Chung et al., 2023a; Markov & Utochkin, 2022; Oberauer, 2019); however, there was reliable evidence against the contribution of conceptual knowledge to VWM precision and/or the number of remembered locations. Nevertheless, meaningful semantics enhanced the capacity of information in VWM. Additional research is required to further uncover the nature of the interaction between LTM and VWM representations.

## Data Availability

Data and materials are available online (https://osf.io/za3bm).

## Footnotes

1 Recently, criticism was raised against some of the theoretical assumptions of mixture-modeling approach (Brady et al., 2024; Schurgin et al., 2020; Williams et al., 2022). Yet not all researchers are convinced by these claims (e.g., Tomić & Bays, 2022). Moreover, the methodological assumptions of the mixture-modeling are met in the present study; radial location is a linear variable (unlike color), and there is sufficient error space for the classification of both swaps and guesses (e.g., Bays, 2016). Furthermore, an empirical model comparison of the data showed that the swap model (Bays et al., 2009) provided the best fit, even against the variable precision model (which holds other theoretical assumptions).
